# The Effects of Lifestyle Interventions on the Health-Promoting Behavior, Type D Personality, Cognitive Function and Body Composition of Low-Income Middle-Aged Korean Women

**DOI:** 10.3390/ijerph18115637

**Published:** 2021-05-25

**Authors:** Eun-Jin Kim, Ju-Hee Nho, Hye-Young Kim, Sook-Kyoung Park

**Affiliations:** 1 College of Nursing, Jeonbuk National University, 567 Baekje-daero, Deokjin-gu, Jeonju 54896, Jeollabuk do, Korea; eunjinKim@jbnu.ac.kr; 2 College of Nursing, Jeonbuk Research Institute of Nursing Science, Jeonbuk National University, 567 Baekje-daero, Deokjin-gu, Jeonju 54896, Jeollabuk do, Korea; tcellkim@jbnu.ac.kr (H.-Y.K.); yoursky@jbnu.ac.kr (S.-K.P.)

**Keywords:** intervention research, health disparities, health behavior, mental health, women’s health, health promotion, health education, middle-aged women, low-income women

## Abstract

Low-income middle-aged women (LMW) who are vulnerable have various physical and psychosocial problems. They need lifestyle interventions to actively cope with these risk factors. This study used a randomized control group pretest-posttest design. LMW aged from 40 to 60 years were recruited and randomly assigned to an experimental group (*n* = 31) and a control group (*n* = 32). The lifestyle interventions for this study, which were implemented for eight weeks, included nutritional management, physical activity, stress management and cognitive function improvement based on King’s goal attainment theory. The measured outcomes were health-promoting behaviors, Type D personality, cognitive function and body composition. The experimental group scored significantly higher than the control group for health-promoting behaviors (effect size (ES) = 0.68~1.27, *p* < 0.001~0.014) and cognitive function (ES = 0.79~1.31, *p* < 0.001~0.005). The negative affectivity (ES = 0.70, *p* = 0.012) and the prevalence of a Type D personality (x^2^ = 4.39, *p* = 0.047) and the systolic blood pressure (ES = 0.65, *p* = 0.019) decreased significantly in the experimental group compared with the control group. Lifestyle interventions for LMW were effective in improving health-promoting behavior, Type D personality traits and cognitive function.

## 1. Introduction

Women aged between 40 and 60 years have a decreased metabolic activity compared with age-matched men. They also experience a disruption of their estrogen-regulating systems as well as depression and impairment in several cognitive areas [1]. The middle age for women is a time when stress can cause a sense of crisis and their health vulnerability increases due to the menopause and aging [2]. There are significant differences in health behavior and the subjective and objective health status among vulnerable low-income women according to their economic level [3] and low-income women have reported having problems with an unhealthy lifestyle [4] and that a low household income negatively affects their health. Moreover, their stress is at its highest when the income level is low [3]. These results suggest the importance of promoting healthy lifestyle behaviors and developing effective health-promoting programs [5]. However, studies on the health status and health behavior of low-income middle-aged women (LMW) are still lacking [3].

Health-promoting activities increase the level of the well-being of individuals and groups and promote self-actualization [6]. As a result, the relationship between the average monthly income and health-promoting behavior (HPB) was confirmed and, hence, a program that could improve these behaviors may be suitable for LMW.

Type D personality comprises two sub-concepts: negative emotions and social restrictions. “D” is characterized by depression, anxiety and worry [7]. According to research, its prevalence is approximately 28% in healthy Korean adults [8] and, of this, 34.5% are Korean middle-aged women. Among the general population and patients with chronic diseases, a Type D personality is vulnerable to stress and is associated with unhealthy behaviors and a low quality of life [9]. Middle-aged women with a Type D personality reported a poorer performance in HPB [10] and it has been reported to be associated not only with cardiovascular diseases but also with psychiatric problems such as anxiety, somatization, posttraumatic stress disorder and depression [11].

Women’s hormonal decline affects the hippocampus, which plays a central role in memory and cognition; therefore, it is associated with neuropathy in the hippocampus, causing cognitive impairment [12]. In a study of 4573 people aged between 50 and 70 years, women showed lower levels of a global cognitive and a domain-specific cognitive function than men, suggesting that intervention strategies for improving cognitive function in women during and after middle age are needed [13].

As such, LMWs show high obesity rates and nutritional imbalances and lifestyle improvements are required to rectify unhealthy dietary habits and irregular exercise [14,15]. In addition, middle-aged women with low incomes have high stress [4] and it was reported that the greater the fear of dementia in middle-aged women, the fewer the health-promoting behaviors. Furthermore, a Type D personality is common among obese and overweight middle-aged women and is related to an unhealthy lifestyle [2]. In many studies, lifestyle interventions (LSIs) including nutrition management, physical activity and stress management were effective in influencing weight control, depression, HPB, reproductive health and the quality of life [2,16,17]. Although personality type is a relatively stable trait, a recent study suggested that LSIs were effective for improving behavior among people with a Type D personality [2]. Physical exercise was found to be a modifiable factor in reducing the risk of dementia [18]. Moreover, brain yoga, which was developed for inducing brain cell activation and neurogenesis based on integrated functional physical exercise, was found to improve short-term memory and increase serum BDNF levels in healthy middle-aged women [19]. As cognitive function declines with age, a physically active lifestyle plays an important role in mitigating this decline [18,20].

Until now, there have been insufficient studies that combine multidimensional factors including cognitive function for LMWs. The interaction between the nurse and the subjects leads to identifying problems and disabilities, setting goals with each other and agreeing on ways to reach the goals. Accordingly, this study aimed to determine the effectiveness of LSIs with an eight-week program based on King’s 1981 goal attainment theory [21] for LMWs. We hypothesized that LMWs who participate in the LSIs would have improved (1) HPB, (2) Type D personality, (3) cognitive function and (4) body composition.

## 2. Materials and Methods

### 2.1. Design

The study adopted a randomized control group (1:1 ratio) pretest-posttest design to examine the effects of LSIs based on King’s theory in LMWs.

### 2.2. Participants

**Figure 1:**
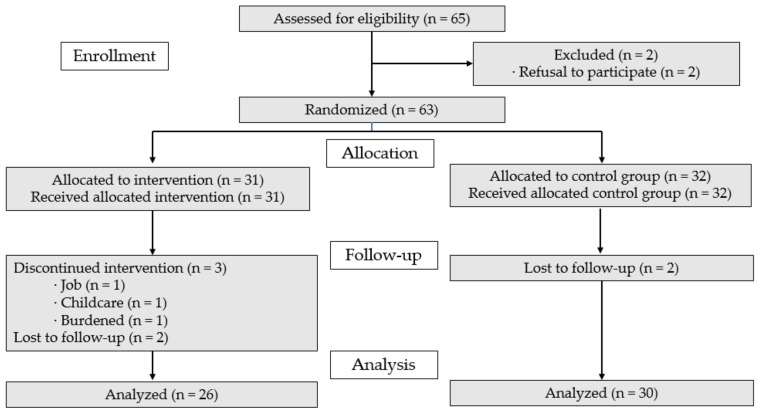
CONSORT flowchart.

A G*Power 3.1.9.2 (IBM Corp., Armonk, NY, USA)statistical analysis was used to determine the sample size for the *t*-test analysis with a significance level of 0.05, power of 0.8 and effect size of 0.75. The effect size and power were calculated based on estimations from previous studies that evaluated the effectiveness of lifestyle interventions [2]. The minimum sample size was 23 per group. A total of 63 women were randomly assigned to the experimental and control groups to account for the 20% dropout rate. A total of 65 women were assessed, two of whom were excluded (*n* = 2, refusal to participate); the 63 final participants were allocated to either the experimental group (*n* = 31) or the control group (*n* = 32). After completing the pretest, five participants in the experimental group declined to participate in the program (job = 1, childcare = 1, burdened = 1, contact loss = 2) while in the control group two participants declined to complete the posttest (contact loss = 2). The dropout rate was 13.9%, resulting in a final total of 56 women; 26 in the experimental group and 30 in the control group (Figure 1). The inclusion criteria were as follows: (ⅰ) women aged 40 to 60 years, (ⅱ) monthly household income below 50% of the national median income and (ⅲ) those who understood the purpose of the research and voluntarily agreed to participate in the research. The exclusion criteria were as follows: (ⅰ) cognitive impairment, (ⅱ) acute heart disease and women taking psychiatric drugs, (ⅲ) women who had received an exercise restriction prescription from a doctor and (ⅳ) women who had participated in other health-related programs within the last three months.

### 2.3. Setting and Data Collection

The participants were recruited from 24 May to 21 September 2020 in J province, South Korea, by using posters that specified the recruitment criteria, advertisements in local newspapers, social networks and posters distributed to related organizations (e.g., community centers, social welfare centers and state-funded educational institutions) along with the contact numbers of the investigator. When the subjects who were interested in participation voluntarily expressed their intention to participate, the researcher assessed whether they met the eligibility criteria. Informed consent was obtained before data collection from those who met the inclusion criteria. The participants were randomly allocated to the experimental or control group using a computer-generated list of random numbers.

### 2.4. Conceptual Framework

In this study, a theoretical framework was constructed based on King’s [21] goal achievement theory. This theory explains the importance of mutual goal setting and the interaction process that leads to goal achievement while the nurse and the subject interact in the environment. A dynamic interaction and the exchange of relationships take place. In this study, the degree of goal achievement was measured to verify the effect of LSIs using the goal achievement theory. In addition, the actions, reactions, mutual setting of goals, search for a goal achievement method, agreement on a goal achievement method, interaction and transaction were included in this study.

First, the action is that the nurse and the subject initiate an action; the nurse proposes a lifestyle program and the LMW suggests their intention to participate in the lifestyle program by applying the goal achievement theory. Second, the reaction to the aforementioned action is where the LMW agrees to participate in the lifestyle program. Third, interaction involves finding specific ways to achieve a goal and agree on a method. Fourth, goals to be achieved are mutually set through the explanation of the importance of the nursing goals to be achieved by the nurse and the subject and to correct and understand negative self-perception and role perception. This aims to improve HPB, decrease the Type D personality, improve cognitive function and improve the body composition index including blood pressure and body mass. Fifth, the search for a goal achievement method is to specifically explore the applicability and understanding of the role of the nurse and the subject considering the situation to achieve the goal. Sixth, the agreement on the method of achieving the goal is one of the methods for achieving the nursing goal for the nurse and the subject. This involves understanding the importance of the need, the specific methods, the benefits to the subject and the importance of trust in nurses and agreeing to the formation of an active relationship, which nurses and the LMW seek and agree on in relation to group and individual interactions. Seventh, the transaction is a step in which the nurse and the subject actively carry out the method agreed upon; a lifestyle program that applies the goal achievement theory is then in progress and individual (between the nurse and the LMW) and group (between LMW groups) transactions occur.

**Figure 2:**
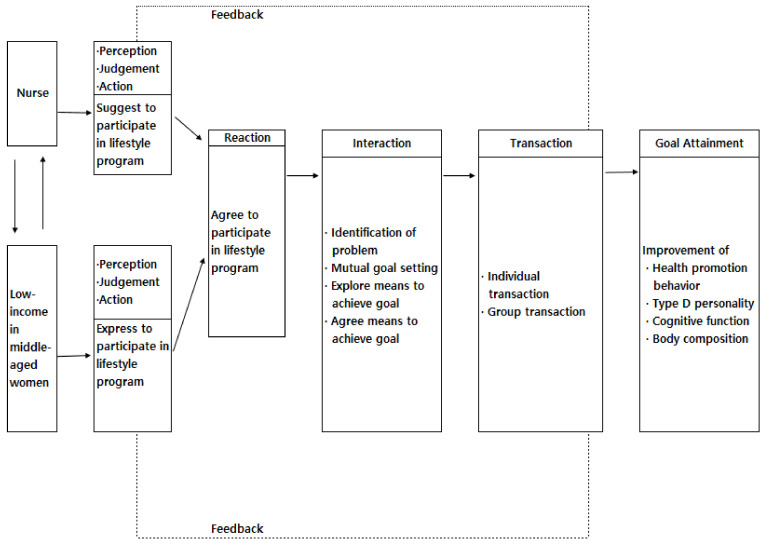
Conceptual framework.

The lifestyle program process through interaction and transaction between the nurses and the LMW shows different behaviors and reactions through feedback, which affects the interaction and transaction between the nurses and the LMW (Figure 2).

### 2.5. Lifestyle Intervention Based on King’s Goal Achievement Theory

We developed an LSI based on King’s goal achievement theory and based on previous literature on LSIs [2,21]. The LSI comprised of eight weekly sessions including four categories of nutritional management, physical activity, stress management and cognitive function improvement [17,19,20]. The nutritional management included eating moderately and regularly, a significant amount of vegetables or fruits and the avoidance of overeating especially at night. Physical exercise included walking more than 10,000 steps per day and exercising for more than 30 min a day to the level of inducing sweating. Stress management is about being satisfied with yourself and having fun. Horticultural therapy improves psychological well-being and quality of life [22]; hence, this study used bouquet-making with fresh flowers for stress management. In addition, “knowing” and “respecting” themselves in the life improvement programs reduced the psychological distress of participants [17]. To improve cognitive function, brain yoga exercise and a dementia prevention finger exercise more than once a day were included along with memorizing phone numbers of important family members and friends and keeping a diet diary. A meta-analysis provided clinicians with the evidence to recommend that patients engage in weekly aerobic and resistance exercise of a moderate intensity [23]. Thus, aerobic muscle strength exercises were performed in every session of this study. In addition, brain yoga is a new form of yoga that helps to improve brain nerve cell activation and production and cognitive ability [19,24,25]. Finger dexterity can reflect a declining cognitive dysfunction and can help to improve cognition [26] (Table 1). The experimental group participated in an eight-session program; the control group was provided with a booklet containing the same dietary advice and exercise contents provided to the experimental group and after the intervention the subjects in the control group were provided with a summary of the LSI if they wanted the full program content.

### 2.6. Outcome Measures

#### 2.6.1. Health-Promoting Lifestyle Profile II (HPLP II)

The HPB was evaluated using the Health-Promoting Lifestyle Profile II (HPLP-Ⅱ) [27]. This instrument has 52 questions consisting of six domains: health responsibility, physical activity, nutrition, spiritual growth, interpersonal relationships and stress management. A higher score indicates a higher level of HPB. The Korean version of the HPLP II has confirmed validity and reliability [28].

#### 2.6.2. Type D Personality

The Type D Scale-14 (DS14), developed by Denollet [29], was used to measure the Type D personality. It consists of seven items for “negative affectivity” and seven for “social inhibition” domains. Each item is scored from 0 to 4 points and a score of 10 or higher on the sub-scales confirms a Type D personality [29]. The Korean version of the DS14 has demonstrated good validity and reliability [8].

#### 2.6.3. Cognitive Function

##### K-MoCA (Korean Version of the Montreal Cognitive Assessment)

Cognitive function was measured using the Korean version of the Montreal Cognitive Assessment (K-MoCA) and the brain-derived neurotrophic factor (BDNF). MoCA evaluates attention-focusing ability, executive function, memory, language ability, poetry composition skills, conceptual thinking, computational ability and mental power [30]. The MoCA is a 30-point test that consists of 32 items where higher scores indicate a better cognition [30]. The Korean version of the MoCA has demonstrated validity and reliability [31].

##### BDNF (Brain-Derived Neurotrophic Factor)

For the BDNF, which can be observed from blood collection, 5 mL of blood was collected from the antecubital vein and coagulated at room temperature for 40 to 60 min and then centrifuged at 3000 rpm for 15 min. The concentration of serum BDNF was analyzed using a standardized sandwich enzyme-linked immunosorbent assay (ELISA) kit (R&D, Minneapolis, MN, USA).

#### 2.6.4. Body Composition

Body composition was assessed according to the body mass index (BMI), body fat (%) and waist–hip ratio. A bioelectrical impedance analysis device (InBody 270) (InBody, Seoul, Korea) was used to investigate the body composition after fasting. The accuracy of this device was 93–96% for the measurement of body water, muscles and fat [32,33]. Blood pressure (BP) was measured using an Omron HEM-7120 (Omron Blood Pressure Monitor, Binh Duong, Vietnam). The demographic factors assessed were age, religion, education, occupation, menopausal status, frequency of exercise and marital status.

### 2.7. Ethical Considerations and Statistical Analysis

The study was approved by the Institutional Review Board of Jeonbuk National University (IRB 2020-02-005-001) of the participating institutions. In addition, this study is registered on the WHO International Clinical Trial Registry Platform (ICTRP; https://cris.nih.go.kr, accessed on 5 May 2021) with the identification number KCT 0006125. In compliance with the Helsinki Declaration, voluntary written consent was obtained after explaining the purpose, content, guarantee of privacy, possibility of opting out and anonymity.

Statistical analyses were performed using SPSS/WIN 25.0 (IBM SPSS Statistics for Windows, IBM Corp., Armonk, NY, USA). The general characteristics of the participants were analyzed using descriptive statistics and the normality test was confirmed using the Shapiro–Wilk test. Depending on the general characteristics of the subjects analyzed in real and percentage, mean and standard deviations, the normality test was confirmed using the Shapiro–Wilk test.

The χ^2^-test, the Fisher exact test, an independent *t*-test and a Mann–Whitney U test were conducted to identify the homogeneity between the experimental and control groups. To analyze the effects of the intervention, the χ^2^-test and independent *t*-test were used. The statistical significance was set at *p* < 0.05.

## 3. Results

### 3.1. Homogeneity Test for General Characteristics and Variables between Groups

There were no significant differences in the general characteristics and variables between the groups at the baseline of those who participated in the final analysis and the allocation (Table 2 and Supplementary Table S1).

### 3.2. Effects of the LSIs

The overall HPLP-II scores in the experimental group were significantly higher than those in the control group (ES = 1.27) and all sub-scales were significantly higher (ES = 0.68~1.10). The score for negative affectivity was significantly lower in the experimental group than in the control group (ES = 0.070) and the prevalence of a Type D personality was significantly lower in the experimental group than in the control group (χ^2^ = 4.39, *p* = 0.047). The K-MoCA score was significantly higher in the experimental group than in the control group (ES = 1.31) and the level of the BDNF was significantly higher (ES = 0.79). The systolic BP in the experimental group was significantly lower than that in the control group (ES = 0.65); however, BMI, body fat, waist–hip ratio and diastolic BP were not significantly different (Table 3).

## 4. Discussion

In this study, after providing LSIs, the HPB significantly improved in the experimental group. The lifestyle appeared to be an HPB internalized by individuals through interactions. This was consistent with studies in which providing LSIs significantly increased the health-promoting behavior of postmenopausal women [34] and increased the effects of HPBs and the physical activity of middle-aged women [20,35]. Taken together, it was believed that the significant increase in HPBs of the LMW in this study was due to continuous counseling by health managers and interventions through HPBs. Meanwhile, the LMW often had to balance household chores and work at the same time so it was difficult to focus on checking their lifestyle habits. Therefore, encouragement and support through continuous exchanges with nurses were effective in improving the health of vulnerable people. In the future, intensive management by health managers, especially nurses, will be required.

The experimental group had a lower prevalence of a Type D personality than the control group. This may be due to the positive effects of meeting with nurses every week to receive nutritional management, health-related counseling, physical activity and stress management feedback. The results of this study were consistent with studies in which HPB improved and the severity of the Type D personality decreased in middle-aged women after LSIs [2]. Furthermore, our results supported the findings of studies demonstrating that a Type D personality has a direct effect on health behavior [11]. Additionally, a Type D personality is associated with an unhealthy lifestyle [10]; patients with a Type D personality are susceptible to health impairments [36] and strategies to reduce a Type D personality are required. Health care providers said that patients with a Type D personality should be provided with ongoing mental health care to ensure that they receive sufficient social support [37]. This study confirmed that LSIs are meaningful in reducing the Type D personality tendency among middle-aged women. Although a Type D personality is a trait, it can be modulated through intervention; therefore, it will be necessary to confirm its effect through LSIs in future studies.

The cognitive function of the experimental group improved more than that of the control group. This was in line with the findings of a study showing that the cognitive function of middle-aged adults improved after an LSI program [38]. Yoga practice is in line with the findings that it can help prevent age-related degeneration by altering heart metabolism risk factors, autonomic function and the BDNF in healthy adults [39]. Moreover, 10 weeks of yoga training increased the BDNF levels and improved motor learning in older adults [40]. Brain yoga was developed using existing integrated functional physical exercise programs as a basis to induce brain cell activation and neurogenesis and improve cognitive functions through yoga [19]. It revealed that a single session of brain yoga significantly improved the short-term memory of middle-aged women and increased serum BDNF levels especially in women without a mild cognitive impairment [19]. Putting these results together, these effects support the claim that the adult brain is profoundly regulated by environmental stimuli to improve cognitive function as exercise restores the proliferating neuron-glial antigen 2 (NG2) cell pool or further increases the proportion of newly differentiated oligodendrocytes [41]. Taken together, the improvement in cognitive function in this study may be due to physical activity along with proper nutrition management. The results confirmed through prior research on LMW in Korea showed poor eating habits, a low physical activity, high stress and low dementia prevention behaviors, which require management [2,3,4,15]. The cognitive function of the participants in this study would have improved because of the nutrition education and physical activity such as brain yoga. Therefore, nutrition management and proper exercise are effective not only for improving the physical function but also for improving the cognitive function.

The systolic blood pressure of the experimental group decreased compared with that of the control group after the interventions. This was in line with a previous study in which the blood pressure was significantly reduced after LSIs [42]. This confirmed that the integrated LSIs conducted in this study were effective in controlling blood pressure, which is a health risk factor. However, long-term interventions will be necessary for the corrected health behavior to become a habit and, therefore, the active interest of nurses and nursing interventions are necessary.

In this study, low-income middle-aged women often had to make a living by doing housework and work at the same time, so it was difficult to focus on checking and practicing their own lifestyle habits. 

The BMI, body fat percentage and waist–hip ratio did not show a statistically significant difference. This was a slightly different result from previous studies showing that after eight weeks of a lifestyle intervention, the body weight decreased by 5.4 ± 4.5 kg and the body fat mass and waist circumference were also decreased [43]. This was presumably because of the limitations in confirming the effects of biochemical indicators. There were also restrictions on outdoor activities due to the COVID-19 pandemic at the time of this study.

This study had several limitations. We used a bioelectrical impedance analysis device to estimate the body composition. Despite the high accuracy of the device, it has limitations in performing qualitative analyses of raw bio-impedance parameters. Campa et al. [44] suggested the general recommendations for a bioelectrical impedance analysis device using a foot-to-hand technique (e.g., device condition, electrode type, environment and time of measurement). In future research, it will be necessary to measure the body composition by considering the recommendations when using a bioelectrical impedance analysis device. The world was affected by COVID-19 and social distancing was required. Under these circumstances, the study proceeded as planned but, as strong social distancing restrictions were implemented in the middle of the eight-week program, the stress of the study participants may also have increased. This study explored the effects before and after the eight-week intervention program but it is necessary to examine the long-term effects to ensure lasting improvements.

## 5. Conclusions

As a result of this study, the HPB of LMW who were provided with LSIs significantly increased. The Type D personality and systolic blood pressure were reduced and cognitive function was also significantly improved. Based on these results, LSIs have been identified as beneficial and can be implemented in community and clinical practice. Our findings can be used as a foundation for future research on LSIs and their long-term health effects.

## Figures and Tables

**Table 1:** Contents of the lifestyle intervention program.

Goal	Session	Contents	Transaction	Minutes
Nutrition management - Moderate and regular meals - Eat a lot of vegetables or fruit - Avoid overeating Physical activity - Walk more than 10,000 steps a day - Exercise for more than 30 min to sweat - Stretch at least twice a day Stress management - Satisfying myself today and being happy - Improve self-esteem Cognitive function improvement - Brain yoga more than once a day - Finger exercise to prevent dementia more than once a day	1	Importance of lifestyle Supply food and lifestyle diary and exercise poster Checking eating habits Physical activity Stretching Muscle strength exercise	Small-group education Face to face encouragement Demonstration and practice Motion counseling Provide exercise video and feedback	60
	2.5	Nutrition management Calorie-based diet for lifestyle, healthy diet The importance of eating vegetables Physical activity Stretching Brain yoga/mat exercise		60
	3.6	Stress management Understanding me Bouquet-making Physical activity Stretching Muscle strength exercise		60
	4.7	Cognitive function improvement Finger exercise to prevent dementia Physical activity Stretching Brain yoga/mat exercise		60
	8	Physical activity Stretching Brain yoga/mat exercise Evaluation of the program		60

**Table 2:** Homogeneity test for participant characteristics and variables in the experimental and control groups (*n* = 56).

Characteristics	Categories	Exp. (*n* = 26)	Con. (*n* = 30)	χ^2^ or *t*	* p *
		* n * (%) or Mean ± SD	* n * (%) or Mean ± SD		
Age (year)		48.19 ± 6.08	50.50 ± 6.35	−1.38	0.172
Religion	Yes	16 (61.5)	19 (63.3)	0.02	>0.999
	No	10 (38.5)	11 (36.7)		
Education	≤Middle school	2 (7.7)	4 (13.3)	0.46 ^a^	0.675
	≥High school	24 (92.3)	26 (86.7)		
Occupation	Yes	10 (38.5)	9 (30.0)	0.45	0.578
	No	16 (61.5)	21 (70.0)		
Menopause	Yes	12 (46.2)	20 (66.7)	2.39	0.177
	No	14 (53.8)	10 (33.3)		
Exercise	No	8 (30.8)	11 (36.7)	1.56	0.460
	Irregular	10 (38.5)	14 (46.7)		
	Regular	8 (30.8)	5 (16.7)		
Marital status	Married	23 (88.5)	27 (90.0)	0.03 ^a^	> 0.999
	Others	3 (11.5)	3 (10.0)		
Variables					
Health-promoting behaviors	Health responsibility	2.51 ± 0.49	2.34 ± 0.64	1.06	0.293
	Physical activity	2.18 ± 0.77	1.98 ± 0.65	1.04	0.301
	Nutrition	2.69 ± 0.52	2.54 ± 0.58	1.02	0.311
	Spiritual growth	2.91 ± 0.66	2.85 ± 0.57	0.40	0.688
	Interpersonal relationship	2.97 ± 0.64	2.99 ± 0.51	−0.10	0.925
	Stress management	2.63 ± 0.50	2.45 ± 0.48	1.41	0.165
	Total	2.65 ± 0.47	2.52 ± 0.44	1.04	0.305
Type D personality	Negative affectivity	7.12 ± 6.00	7.80 ± 4.78	−0.48	0.637
	Social inhibition	7.23 ± 6.11	7.37 ± 4.83	−0.09	0.926
	Type D	6 (23.1)	8 (26.7)	0.10	>0.999
	Non-Type D	20 (76.9)	22 (73.3)		
Cognitive function	K-MoCA	24.58 ± 1.78	24.17 ± 1.49	0.94	0.350
	BDNF (pg/mL)	26,327.33 ± 7174.28	26,157.77 ± 7186.30	0.09	0.930
Body composition	BMI (kg/m^2^)	23.83 ± 4.06	23.50 ± 3.39	0.33	0.746
	Body fat (%)	33.10 ± 5.55	32.64 ± 5.76	0.31	0.760
	Waist–hip ratio (%)	0.86 ± 0.05	0.88 ± 0.04	−1.65	0.104
	SBP (mmHg)	128.58 ± 13.65	124.17 ± 16.21	1.09	0.280
	DBP (mmHg)	83.73 ± 10.43	81.17 ± 10.93	0.89	0.375

BDNF, brain-derived neurotrophic factor; BMI, body mass index; Con., control group; DBP, diastolic blood pressure; Exp., experimental group; K-MoCA, Korean-Montreal Cognitive Assessment; SBP, systolic blood pressure; ^a^ Fisher’s exact test.

**Table 3:** Effects of the lifestyle interventions (*n* = 56).

Variables	Characteristics	Group	Pretest	Posttest	Differences	χ^2^ or *t*	* p *	ES (d)	Power
			Mean ± SD	Mean ± SD	Mean ± SD				
Health promoting behaviors	Health responsibility	Exp.	2.51 ± 0.49	2.97 ± 0.41	0.46 ± 0.54	3.74	<0.001	1.01	0.98
		Con.	2.34 ± 0.64	2.28 ± 0.56	−0.07 ± 0.51				
	Physical activity	Exp.	2.18 ± 0.77	2.83 ± 0.48	0.64 ± 0.60	4.10	<0.001	1.10	0.99
		Con.	1.98 ± 0.65	1.94 ± 0.56	−0.05 ± 0.65				
	Nutrition	Exp.	2.69 ± 0.52	3.05 ± 0.50	0.36 ± 0.47	3.09	0.003	0.82	0.92
		Con.	2.54 ± 0.58	2.51 ± 0.54	−0.03 ± 0.48				
	Spiritual growth	Exp.	2.91 ± 0.66	3.18 ± 0.40	0.27 ± 0.61	2.53	0.014	0.68	0.81
		Con.	2.85 ± 0.57	2.71 ± 0.63	−0.14 ± 0.59				
	Interpersonal relationship	Exp.	2.97 ± 0.64	3.18 ± 0.45	0.20 ± 0.47	3.10	0.003	0.83	0.92
		Con.	2.99 ± 0.51	2.77 ± 0.59	−0.22 ± 0.54				
	Stress management	Exp.	2.63 ± 0.50	3.05 ± 0.46	0.42 ± 0.47	3.96	<0.001	1.06	0.99
		Con.	2.45 ± 0.48	2.35 ± 0.57	−0.09 ± 0.49				
	Total	Exp.	2.65 ± 0.47	3.04 ± 0.37	0.39 ± 0.39	4.73	<0.001	1.27	0.99
		Con.	2.52 ± 0.44	2.43 ± 0.48	−0.10 ± 0.38				
Type D personality	Negative affectivity	Exp.	7.12 ± 6.00	5.54 ± 4.61	−1.58 ± 3.94	−2.59	0.012	0.70	0.83
		Con.	7.80 ± 4.78	9.37 ± 5.74	1.57 ± 4.99				
	Social inhibition	Exp.	7.23 ± 6.11	5.73 ± 3.99	−1.50 ± 5.93	−1.99	0.052	0.53	0.62
		Con.	7.37 ± 4.83	8.67 ± 4.48	1.30 ± 4.59				
	Type D	Exp.	6 (23.1)	2 (7.7)		4.39	0.047		
		Con.	8 (26.7)	9 (30.0)					
	Non-Type D	Exp.	20 (76.9)	24 (92.3)					
		Con.	22 (73.3)	21 (70.0)					
Cognitive function	K-MoCA	Exp.	24.58 ± 1.78	27.00 ± 1.44	2.42 ± 1.86	150.00 ^a^	<0.001	1.31	0.99
		Con.	24.17 ± 1.49	23.57 ± 2.60	−0.60 ± 2.74				
	BDNF (pg/mL)	Exp.	26,327.33 ± 7174.28	30,780.13 ± 8466.98	4452.80 ± 5377.10	2.95	0.005	0.79	0.89
		Con.	26,157.77 ± 7186.30	26,787.33 ± 7043.10	629.56 ± 4333.05				
Body composition	BMI (kg/m^2^)	Exp.	23.83 ± 4.06	23.64 ± 3.97	−0.19 ± 0.52	318.00	0.232	0.39	0.42
		Con.	23.50 ± 3.39	23.51 ± 3.37	0.01 ± 0.50				
	Body fat (%)	Exp.	33.10 ± 5.55	32.34 ± 5.90	−0.76 ± 2.31	343.00 ^a^	0.412	0.53	0.62
		Con.	32.64 ± 5.76	32.63 ± 5.60	−0.01 ± 0.50				
	Waist–hip ratio (%)	Exp.	0.86 ± 0.05	0.88 ± 0.06	0.01 ± 0.03	313.50 ^a^	0.243	0.33	0.34
		Con.	0.88 ± 0.04	0.89 ± 0.05	0.00 ± 0.02				
	SBP (mmHg)	Exp.	128.58 ± 13.65	117.19 ± 12.28	−11.38 ± 11.59	−2.42	0.019	0.65	0.78
		Con.	124.17 ± 16.21	121.03 ± 13.71	−3.13 ± 13.62				
	DBP (mmHg)	Exp.	83.73 ± 10.43	75.65 ± 8.98	−8.08 ± 8.87	308.00 ^a^	0.180	0.41	0.45
		Con.	81.17 ± 10.93	77.13 ± 9.83	−4.03 ± 10.92				

BDNF, brain-derived neurotrophic factor; BMI, body mass index; Con., control group; Exp., experimental group; DBP, diastolic blood pressure; ES, effect size; K-MoCA, Korean-Montreal Cognitive Assessment; SBP, systolic blood pressure. Lifestyle interventions provided before and after the test results to the independent *t*-test for the difference before and after intervention to measure the changes in health-promoting behaviors applied before and after the intervention group difference 0.39 ± 0.39, control group −0.10 ± 0.38 in the control group than life. ^a^ Mann–Whitney U test.

## Data Availability

The data presented in this study are available on request from the corresponding author. The data are not publicly available due to privacy.

## Supplementary Materials

The following are available online at https://www.mdpi.com/article/10.3390/ijerph18115637/s1, Table S1: Homogeneity test for participant characteristics and variables in the experimental and control groups at allocation (*N* = 63).
