# Supplementary material for: The Effects of Lifestyle Interventions on the Health-Promoting Behavior, Type D Personality, Cognitive Function and Body Composition of Low-Income Middle-Aged Korean Women
Source: Int J Environ Res Public Health. 2021 May 25;18(11):5637. doi: 10.3390/ijerph18115637 (PMC8197549; doi:10.3390/ijerph18115637)
Supplement: Supplementary file 1 [file ijerph-18-05637-s001.zip › ijerph-1199244-supplementary.pdf]

International Journal of
Environmental Research and Public Health

Supplementary Table S1. Homogeneity test for participant characteristics and variables in the experimental and control groups at allocation (N=63).

Characteristics	Categories	Exp. (n=31)	Con. (n=32)	χ^2 or t	*p*
		n(%) or Mean±SD	n(%) or Mean±SD		
Age (year)		47.87±5.89	50.06±6.41	-1.41	.163
Religion	Yes	20(51.3)	19(48.7)	0.18	.674
	No	11(45.8)	13(54.2)		
Education	≤ Middle school	2(28.6)	5(71.4)	1.34[a]	.426
	≥ High school	29(51.8)	27(48.2)		
Occupation	Yes	13(56.5)	10(43.5)	0.78	.378
	No	18(45.0)	22(55.0)		
Menopause	Yes	14(41.2)	20(58.8)	1.91	.167
	No	17(58.6)	12(41.4)		
Exercise	No	9(45.0)	11(55.0)	0.93	.629
	Irregular	13(46.4)	15(53.6)		
	Regular	9(60.0)	6(40.0)		
Marital status	Married	28(50.0)	28(50.0)	0.13[a]	.722
	Others	3(42.9)	4(57.1)		
Variables					
Health-promoting behaviors	Health responsibility	2.50±0.51	2.35±0.63	1.00	.320
	Physical activity	2.14±0.80	1.99±0.63	0.82	.416
	Nutrition	2.68±0.52	2.56±0.57	0.86	.392
	Spiritual growth	2.99±0.67	2.83±0.56	1.02	.310
	Interpersonal relationship	3.00±0.62	2.98±0.51	0.15	.310
	Stress management	2.64±0.53	2.46±0.47	1.43	.157
	Total	2.16±0.40	2.03±0.37	1.29	.201
Type D personality	Negative affectivity	7.38±5.99	7.75±4.80	-0.27	.791
	Social inhibition	7.32±5.95	7.50±4.75	-0.13	.896
	Type D	8(47.1)	9(52,9)	0.05	.836
	Non-Type D	23(50.0)	23(50.0)		
Cognitive function	K-MoCA	24.52±1.65	24.13±1.45	0.99	.322
	BDNF (pg/ml)	26953.03±6841.02	26265.79±7089.79	0.39	.697
Body composition	BMI (kg/m²)	24.27±4.14	23.43±3.40	0.87	.387
	Body fat (%)	33.92±6.10	32.50±5.73	0.95	.345
	Waist-hip ratio (%)	0.87±0.05	0.88±0.04	-.98	.331
	SBP (mmHg)	128.16±13.12	124.50±16.00	0.99	.325
	DBP (mmHg)	82.94±9.90	80.78±10.83	0.82	.414

Note. BDNF, brain-derived neurotrophic factor; BMI, body mass index; Con., control group; DBP, diastolic blood pressure; Exp., experimental group; K-MoCA, Korean-Montreal Cognitive Assessment; SBP, systolic blood pressure; [a] Fisher`s exact test.
